# Exercise Therapy Augments the Ischemia-Induced Proangiogenic State and Results in Sustained Improvement after Stroke

**DOI:** 10.3390/ijms14048570

**Published:** 2013-04-18

**Authors:** Yuewen Ma, Lin Qiang, Man He

**Affiliations:** Department of Rehabilitation Medicine, The First Affiliated Hospital, China Medical University, Shenyang 110001, Liaoning, China; E-Mails: qianglin@yeah.net (L.Q.); zhanglucydai@126.com (M.H.)

**Keywords:** treadmill training, stroke, vascular endothelial growth factor, matrix metalloproteinase 2, bevacizumab

## Abstract

The induction of angiogenesis will stimulate endogenous recovery mechanisms, which are involved in the long-term repair and restoration process of the brain after an ischemic event. Here, we tested whether exercise influences the pro-angiogenic factors and outcomes after cerebral infarction in rats. Wistar rats were exposed to two hours of middle-cerebral artery occlusion and reperfusion. Different durations of treadmill training were performed on the rats. The expression of matrix metalloproteinase 2 (MMP2) and vascular endothelial growth factor (VEGF)-related genes and proteins were higher over time post-ischemia, and exercise enhanced their expression. Sixteen days post-ischemia, the regional cerebral blood flow in the ischemic striatum was significantly increased in the running group over the sedentary. Although no difference was seen in infarct size between the running and sedentary groups, running evidently improved the neurobehavioral score. The effects of running on MMP2 expression, regional cerebral blood flow and outcome were abolished when animals were treated with bevacizumab (BEV), a VEGF-targeting antibody. Exercise therapy improves long-term stroke outcome by MMP2-VEGF-dependent mechanisms related to improved cerebral blood flow.

## 1. Introduction

Exercise therapy is a well-known component of stroke rehabilitation programs [1–3]. After stroke, the newborn neuroblasts migrate along peri-vascular routes [4], and promotion of angiogenesis stimulates endogenous recovery mechanisms [5–8]. Exercise preconditioning enhances cerebrovascular integrity [9] and improves angiogenesis and cerebral blood flow [10] in ischemic rats. Hence, clarifying the molecular mechanisms by which exercise exerts its functions in neurovascular remodeling may offer a potential breakthrough for the development of new methods that improve long-term outcome after stroke.

Many studies have shown that the vascular endothelial growth factor (VEGF) expression was induced in experimental brain ischemia [11–16]. It has become clear that VEGF is important in the hypoxic initiation of endothelial cell proliferation and migration that are required for angiogenesis to occur [17]. When VEGF signaling was blocked with a receptor inhibitor, recruitment of new neurons was impaired [18]. To accommodate the migration of the proliferating cells, a finely regulated degradation of the basement membrane and surrounding extracellular matrix (ECM) must occur. Clearance of the basement membrane prior to release and migration requires the production of ECM-degrading proteinases. One family of such enzymes is the matrix metalloproteinases (MMPs), which are zinc proteases that cleave components of the ECM. The MMPs include three major types: the collagenases, the stromelysins and the gelatinases [19]. They protracted pathophysiological cellular remodeling that is triggered by ischemia [20]. An ischemic episode elicits upregulation of matrix metalloproteinase 2 (MMP2), MMP3, MMP7, MMP9 and MMP14 by endothelial cells, pericytes, leukocytes and other components of the neurovascular epithelium [21]. Several MMPs are believed to be important in angiogenesis, but particular interest has been focused on the MMP2 and MMP9, since they preferentially degrade basement membrane components, such as type IV collagen [22]. Although collagen is not a dominant feature of the extracellular matrix in the brain and is only found around the cerebrovasculature. Cell culture assays demonstrate that myelin-associated glycoprotein can be cleaved by MMP2, releasing bioactive fragments that inhibit axonal outgrowth from dorsal root ganglion neurons [23,24]. Inhibition of VEGF downregulated visfatin-induced MMP2 induction and endothelial angiogenesis, as evidence for the relationship of these two pro-angiogenic factors [25]. Exercise increases MMP2 expression in skeletal muscles and the ECM of the muscle [26,27].

With the aforementioned, this study was designed to test the hypothesis that regular exercise therapy augments regional cerebral blood flow (rCBF), thereby improving long-term recovery in a well-characterized rat model of mild stroke. In order to further clarify the molecular background to this condition, we evaluated dynamic changes in the key pro-angiogenic factors VEGF and MMP2, and examined the role of VEGF in regulating the levels of MMP2.

## 2. Results and Discussion

### 2.1. Neurobehavioral Scores

**Figure 1:**
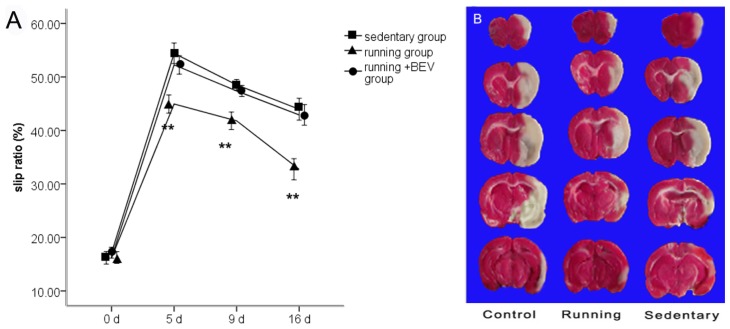
(**A**) The slip ratio of hind limbs in the beam-walking test. The data are expressed as slips ratio (made slips/steps) for the impaired (contralateral to lesion) hind limb. The data (*n* = 5) are the mean ± S.E.M. **p < 0.05 compared to the sedentary group; (**B**) This photograph shows a representative cerebral infarct of the brain slices in the sham, running and sedentary groups at 16 days. The pale region is the infarct brain tissue and the red region is normal tissue. The infarct volume of five rats in the sham group was zero (not shown). There was no statistically significant difference between the indicated running group and sedentary group.

Running conferred sensory-motor deficit scores, there was significant improvement in running compared with sedentary rats (Figure 1A). Bevacizumab (BEV) significantly weakened the protective effect of physical activity (Figure 1A). Average blood pressure (BP) and heart rate (HR) were not different in 16-days running (diastolic BP, 93 ± 14 mm Hg; mean arterial BP, 105 ± 13 mm Hg; systolic BP, 117 ± 29 mm Hg; HR, 439 ± 48 bpm) compared with sedentary (diastolic BP, 92 ± 11 mm Hg; mean arterial BP, 103 ± 14 mm Hg; systolic BP, 125 ± 35 mm Hg; HR, 485 ± 57 bpm; *p* > 0.05; *n* = 5 per group). Physiological parameters did not differ significantly between running *versus* sedentary animals (pH, 7.30 ± 0.05 *versus* 7.28 ± 0.03; PCO_2_, 53 ± 2 *versus* 48 ± 4 mm Hg; PO_2_, 85 ± 8 *versus* 89 ± 5 mm Hg in sedentary *versus* running; *p* > 0.05; *n* = 5 per group).

### 2.2. Infarct Volume

Temporary MCAo/R produced histological damage in the cortex and striatum in the MCAo/R groups, with no significant difference between the sedentary group (45.6% ± 2.3%) and running group (43.7% ± 3.8%) after two weeks of treadmill training (Figure 1B).

### 2.3. Running Enhances MMP2 and VEGF mRNA Levels

**Figure 2:**
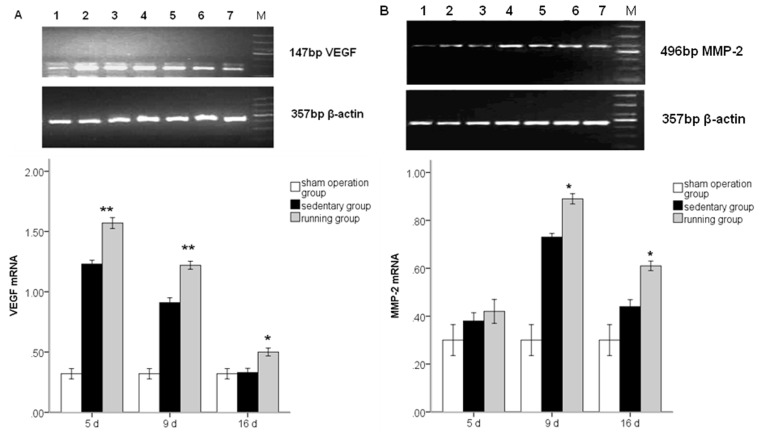
Vascular endothelial growth factor (VEGF) (**A**) and matrix metalloproteinase 2 (MMP2) (**B**) mRNA expression in peripheral ischemia over time after MCAo/R injury. The experimental groups are represented by the numbers: 1, sham group; 2, running five-day group; 3, sedentary five-day group; 4, running nine-day group; 5, sedentary nine-day group; 6, running 16-day group; 7, sedentary 16-day group; M, marker. The data (*n* = 5) are the mean ± S.E.M.*p < 0.05, **p < 0.01 compared to the sedentary group.

The angiogenic potential is greatly enhanced by the degradation of the ECM, where gelatinase MMP2 and VEGF play a vital role. A more intense expression of the gene for VEGF was indicated in MCAo/R sedentary group than in sham control as early as five days post-stroke and then declined thereafter. Enhanced MMP2 mRNA was observed at nine days, later than VEGF. At five days, running induced a significant increase in mRNA expressions of VEGF, and at nine days, MMP2 mRNA was also higher in running group than sedentary (Figure 2A,B, *p* < 0.01).

### 2.4. Effect of Running on MMP2 and VEGF Protein Expressions and the Effect of BEV

**Figure 3:**
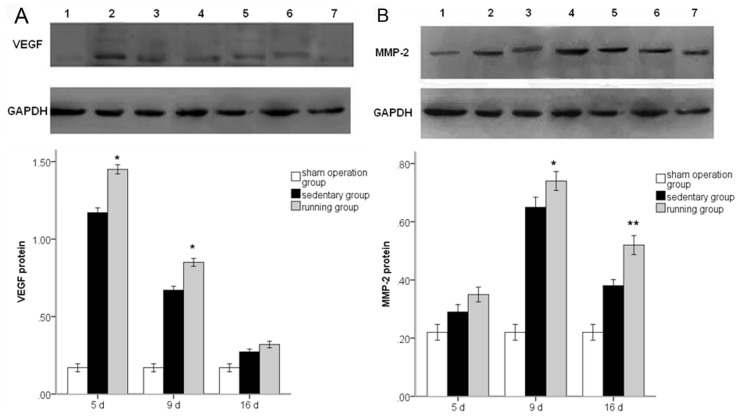
VEGF (**A**) and MMP2 (**B**) protein expression in peripheral ischemia over time after MCAo/R injury. The experimental groups are represented by the numbers: 1, sham group; 2, running five-day group; 3, sedentary five-day group; 4, running nine-day group; 5, sedentary nine-day group; 6, running 16-day group; 7, sedentary 16-day group. The data (*n* = 5) are the mean ± S.E.M. *p < 0.05, **p < 0.01 compared to the sedentary group.

**Figure 4:**
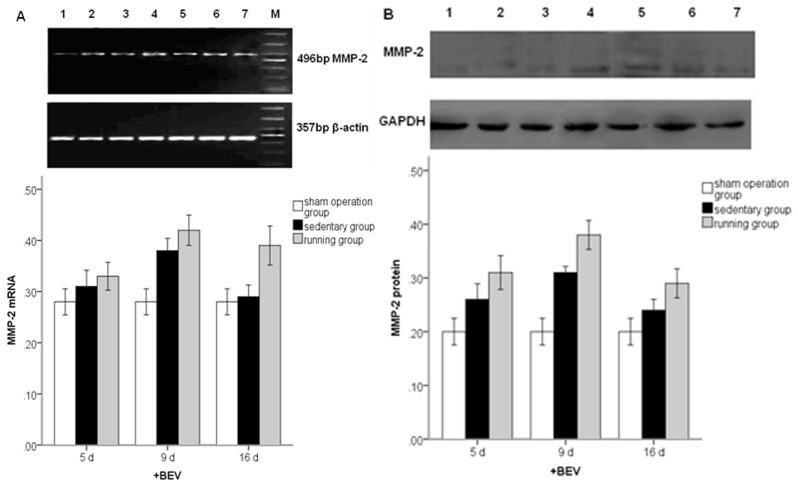
MMP2 mRNA (**A**) and protein (**B**) expression in peripheral ischemia over time co-treated with BEV after MCAo/R. The experimental groups are represented by the numbers: 1, sham group; 2, running five-day group; 3, sedentary five-day group; 4, running nine-day group; 5, sedentary nine-day group; 6, running 16-day group; 7, sedentary 16-day group; M, marker. The data (*n* = 5) are the mean ± S.E.M.

To further quantify and confirm the effect of running on the levels of VEGF and MMP2, protein expression was assayed by Western blot analysis. Examination of concentrations of VEGF and MMP2 in the running and sedentary groups confirmed the RT-PCR observation (Figure 3A,B, *p* < 0.01). The expression of MMP2 reached a peak later than VEGF. We infer that VEGF may have some effect on the ischemic-induced expression of MMP2 and the running-induced augmentation. BEV treatment blunted the MMP2 stimulation observed after MCAo/R both in the running and sedentary group (Figure 4A,B).

### 2.5. Running Increases rCBF

**Figure 5:**
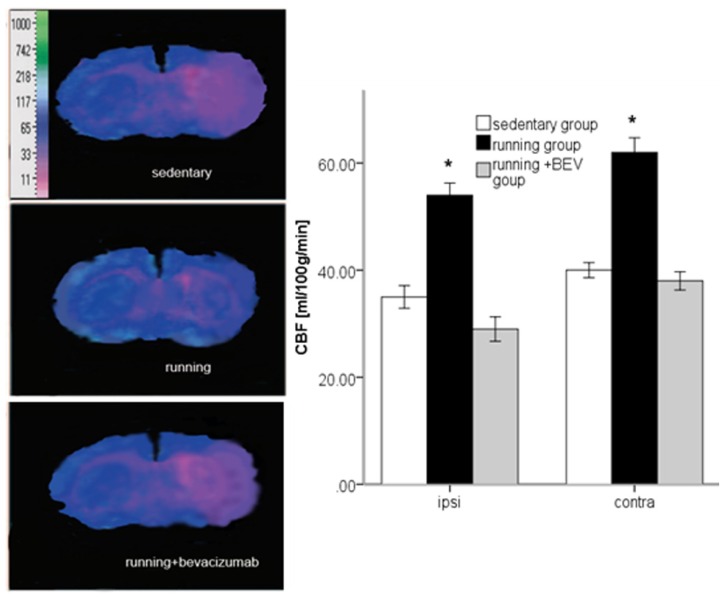
After two hours of MCAo/reperfusion or sham operation, Wister rats performed two weeks of voluntary running or a sedentary life-style or running co-treated with BEV. At 16 days after MCAo/r, absolute cerebral blood flow (CBF) was determined in the striatum of contralateral and ischemic hemispheres using the 14C-iodoantipyrine tissue equilibration technique. Representative pseudocolored autoradiographic images of coronal brain sections are shown. The data (*n* = 5) are the mean ± S.E.M.*p < 0.05, **p < 0.01 compared to the sedentary group. No significant CBF changes were detected in sham-operated animals (*n* = 5).

Absolute rCBF was significantly lower in the ischemic striatum compared with contralateral side in sedentary rats at three weeks (Figure 5). In runners, the rCBF was significantly higher both in the ischemic and contralateral striatum compared with sedentary animals, an effect that was significantly weakened after BEV co-treatment (Figure 5). In sham-operated rats, we detected no significant CBF alterations (data not shown). In addition, the results also showed that BEV can potentially cross the BBB in the stroke area, and the CBF was hampered in contralateral hemisphere by BEV administration.

### 2.6. Discussion

It is now appreciated that angiogenesis will stimulate endogenous recovery mechanisms following brain ischemia, including neurogenesis, synaptogenesis and neuronal and synaptic plasticity. Dysregulated angiogenesis involves the VEGF-MMP system [28]. Although inhibition of endogenous growth factors seems beneficial in certain ophthalmic conditions, atherosclerotic plaques, tumor and so on, extending diametrically opposed therapeutic advancements is necessary in inducing angiogenesis for improving vascular insufficiency [29–31]. Therefore, the study of VEGF-MMP system assumes crucial importance. In this study, we have linked the beneficial effects of regular physical activity on VEGF-MMP and absolute rCBF to long-term recovery following brain ischemia.

In the present study, we conducted a well-characterized mouse model of focal cerebral ischemia and found that brain ischemia upregulated VEGF and MMP2 expression, which is further enhanced by treadmill training. In addition, we reported that running significantly augmented absolute rCBF in the ischemic lesion 16 days after stoke. The proangiogenic effects of running are associated with improved outcome, as evidenced by better sensory-motor deficit scores. There were no apparent changes in any physiological parameter that could explain these effects. More importantly, we demonstrated the role of VEGF signaling in exercise-induced MMP2 upregulation and increased absolute rCBF, a role that appeared to mediate the protective effects of regular physical activity. As it was revealed that when animals were co-treated with BEV, the running effects on MMP2 upregulation, rCBF and outcome were apparently weakened, a finding indicated that increased VEGF bioavailability may stimulate MMP2 expression. Previous literature demonstrated that when animals were trained only after ischemia, there was a nonsignificant trend toward smaller lesions [32], and our results confirmed this notion.

The MMPs are increasingly being recognized as having beneficial roles following nervous system injury. They are involved in endogenous mechanisms of neurogenic migration as the brain seeks to heal itself after ischemic injury [33,34]. The MMP proteolysis of ECM molecules may perform a permissive or inductive role in fiber remodeling and synaptogenesis initiated by deafferentation [35]. The MMP-mediated breakdown of the brain blood barrier (BBB), which occurs over the course of 24–48 h, can trigger neuronal apoptosis and synapse loss. After this protracted destructive phase, further MMP expression can contribute to subsequent reparative processes of cell replacement, remyelination and reestablishment of connectivity and neurovascular integrity [20,21,36,37]. In this study, brain ischemia induced a significantly higher expression of MMP2 at nine days post-ischemia, a little different from some previous studies, which demonstrated that MMP2 activity was maximum at five days after MCAo around the ischemic core [38–40]. Furthermore, treadmill training significantly augmented the expression of MMP2 after training for seven days and 14 days, and rCBF was elevated in the running group 16 days after MCAo/R. However, for the 16-days sedentary rats, this angiogenic response was apparently abortive. It was speculated that the later expression of MMP2 by macrophages may aid in their migration into the ischemic lesion and contribute to the clearing of cellular debris during the later wound-healing/resolution phase after focal stroke [39]. We have shown the effect of running on the VEGF expression in the ischemic brain tissue besides MMP2. The proangiogenic effects of running are associated with improved outcome. Collectively, our findings would tentatively support that the time of appearance of MMP2, as well as its augmentation by running, may be involved in tissue repair [38] and contribute to the remodeling of the white matter myelin and microvascular beds in chronic cerebral hypoperfusion [40]. Exercise therapy after cerebral ischemia stroke increases the expression of MMP2 and VEGF, which play a positive role in neurological recovery. It is conjectured that, through promoting angiogenesis and nerve regeneration, both VEGF and MMP2 can increase ischemic brain tissue oxygen supply and promote brain remodeling, so as to promote the recovery of neural function.

A feature of early vascular remodeling is enhanced production of MMPs, in particular, MMP2 [41–44]. The MMPs are regulated by a number of cytokines and growth factors [45]. Several previous studies have reported that VEGF can induce reduction of infarct size [46–48], promote ischemia-induced neurogenesis and stimulate angiogenesis in the ischemic rat brain [49,50]. Lamoreaux suggested that VEGF may modulate endothelial cell-derived MMP activity by increasing the abundance of MMP2 and disinhibiting MMP2 by decreasing the abundance of an endogenous MMP inhibitor, a tissue inhibitor of metalloproteinase-2 (TIMP-2) [51]. These actions could contribute to the ability of VEGF to promote endothelial cell invasion of new territory. In this experiment, the expression of VEGF increased for peak at five days after cerebral ischemia. Moreover, we provided novel evidence that running-induced MMP2 expression were significantly reduced when co-treated with BEV, a VEGF targeting antibody. This observation was further extended to running-induced higher rCBF and better functional outcome, both weakened by BEV. In fact, MMP2 activity is increased in several types of endothelial cells treated with VEGF or exposed to hypoxia [52]. It is therefore likely that an increase in MMP2 activity is necessary for endothelial cell release from the basement membrane, as well as for invasion into new territories during angiogenesis. One of the roles of VEGF following its release would be to modulate the activities of MMP2, which would presumably allow the endothelial cells to obtain a path by which to migrate, eventually forming a capillary sprout, vessel cord and, finally, a capillary network. Because of the instructive effects of angiogenesis during neurological recovery after stroke, the regulation of VEGF and MMP2 activity offers a means of stimulating the angiogenic response and could therefore be a target of pharmacological activation of angiogenesis. It should be emphasized that the rCBF, as an indicator for tissue perfusion, reflect both microvessel density and caliber. The substances that improve vasodilation, for example nitric oxide, can alter the results.

## 3. Experimental Section

### 3.1. Animals and Treatment

All experimental procedures that were performed on laboratory animals conformed to the Guidelines for Animal Experimentation of the Institutional Animal Care and Use Committee of China Medical University. Ninety adult male Wister rats (250–350 g, clean animals, Animal Department of China Medical University) were housed with free access to food and water and placed on a 12/12 h light/dark cycle. Rats were first familiarized with the treadmill and then randomly assigned to ten groups (Table 1). The treadmill training-treated animals were exercised on a four-lane treadmill (JX-240 treadmill, Xuzhou Fitness Equipment Company, Xuzhou, China) at 3 days after MCAo/R at a speed of 12 m/min for 30 min each day. The rats were trained for 3 days, 7 days and 14 days, respectively, in the 5-days, 9-days and 16-days after MCAo/R running groups. The BEV co-treated animals received bevacizumab (Avastin; Genentech/Roche, San Francisco, CA, USA), 45 mg/kg *i.v*., immediately after reperfusion.

### 3.2. Cerebral Ischemia and Measurement of Physiological Parameters

The MCAo/R-treated rats were anesthetized with 1 vol% isoflurane in 69% N_2_O and 30% O_2_ and subjected to right MCAo for 2 h followed by reperfusion, as described [9]. The sham-operated rats underwent the same surgical procedure, except that the polyester thread was advanced into the internal carotid artery (ICA) for 9 mm. Core temperature was maintained at 36.5 ± 0.5 °C. The neurological deficits were scored using a modified scoring system based on the system developed by Longa *et al.*[53]: 0, no deficits; 1, difficulty in fully extending the contralateral forelimb; 2, unable to extend the contralateral forelimb; 3, mild circling to the contralateral side; 4, severe circling; and 5, falling to the contralateral side. Three animals died after MCAo/R, and the same number of animals was supplemented. The rats with a score of 2–4 were included in the experimental group. In five rats from 16-days MCAo/R running group and five from 16-days MCAo/R sedentary, the left femoral artery was cannulated. Arterial blood samples were analyzed for blood gases, as described [54]. In addition, for telemetric recording, a telemetry probe (TA11PA-C-20, Data Sciences International, St. Paul, MN, USA) was inserted and secured in the right common carotid artery, and the transmitter was placed subcutaneously. After a 10-day recovery period, blood pressure, heart rate and physical activity were continuously measured for 72 h (all parameters plus waveform files were recorded for 1 min every 10 min). The data were analyzed with the A.R.T.2.1 software (Data Sciences International).

### 3.3. Neurobehavioral Tests

The tapered/ledged beam tests selected for the study are sensitive for the detection of long-term impairment in sensory-motor functions [55]. The animals were tested before MCAo and on post-MCAo/R days 5, 9 and 16 before sacrifice. All behavioral analyses were performed in a blinded manner. The rats were pre-trained for 3 days to traverse the beam before ischemia induction. The beam-walking apparatus consisted of a tapered beam with under-hanging ledges on each side to permit foot faults without falling. The end of the beam was connected to a black box (20.5 × 25 × 25 cm^3^) with a platform at the starting point. A bright light was placed above the start point to motivate the rats to traverse the beam. Each rat’s performance was videotaped and then analyzed by calculating the slip ratio of the impaired (contralateral to lesion) hind limb: more slips indicated a greater degree of impairment. Steps onto the ledge were scored as a full slip, and a half slip was scored if the limb touched the side of the beam. The slip ratio was calculated as follows: [(number of full slips + 0.5 × number of half slips)/(number of total steps)] × 100%. The mean of three trials was used for the statistical analysis.

### 3.4. Measurement of Cerebral Infarction Volume

Five rats from the MCAo/R control group, five from the 16-days running and five from the 16-days sedentary were euthanized after neurobehavioral tests with chloral hydrate, and the brains were collected quickly and placed at −20 °C for 30 min. The brain tissue was cut into 5 coronal sections 3 mm thick and stained with a 2% solution of triphenyl tetrazolium chloride (TTC) in PBS at a temperature of 37 °C for 20 min, followed by 4% paraformaldehyde buffer for fixation. The stained sections were photographed, and the digital images were analyzed using imaging software (Adobe Photoshop 7.0) (the pink area was normal brain tissue, and the pale area indicated infarction). The total infarction volume was calculated as the sum of the area of the brain infarction multiplied by the thickness of each section (3 mm). The lesion volumes were calculated by the following formula: {[total infarct volume − (the volume of the intact ipsilateral hemisphere − the volume of the intact contralateral hemisphere)]/contralateral hemisphere volume} × 100%. This indirect measure corrects for edema in the total infarct volume.

### 3.5. Reverse-Transcription Polymerase Chain Reaction

Immediately after the tapered/ledged beam tests, the rats were sacrificed by decapitation, and right brain tissues between the anterior and posterior fontanel were dissected and frozen. The brain tissues were equally divided into 2 parts; the anterior parts were used for extracting RNA and the posterior for Western blot analysis.

The Reverse transcription polymerase chain reaction (RT-PCR) technique was used to determine the expression of genes encoding MMP2 and VEGF. Total RNA was isolated using a reverse transcription kit (RNAiso™ PLUS, Takara, Otsu, Japan). We obtained cDNA by reverse transcription, which was performed using a reverse transcription kit (Takara, Otsu, Japan), according to the manufacturer’s protocol. The amplification was performed as follows: for MMP2, VEGF and β-actin, 35 cycles of predenaturation at 94 °C for 2 min, denaturation at 94 °C for 30 s, annealing at 58 °C for 30 s and elongation at 72 °C for 30 s. The PCR primers were as follows: MMP2 (496 bp), 5′-GCAACCACAACCAACTACGAT-3′ (forward) and 5′-CATTCCCTGCGAAGAACACA-3′ (reverse); VEGF (147 bp), 5′-GGACATCTTCCAGGAGTACC-3′ (forward) and 5′-CGCATGATCTG CCATAGTGCA-3′ (reverse); β-actin (357 bp), 5′-TAAAGACCTCTATGCCAACAC-3′ (forward) and 5′-TAAAGCCATGCCTAATGTCTC-3′ (reverse). The amplified samples were subjected to electrophoresis through 2% agarose gels. The fluorescence intensity of each band was scanned and quantified using NIH-image software. The levels of MMP2 and VEGF were normalized against those of β-actin mRNA.

### 3.6. Western Blot Analysis

The total protein of the frozen specimens was extracted by a commercial protein extraction reagent (Pierce, Rockford, IL, USA) at a ratio of 1 g of tissue to 10 mL reagent, according to the product manual. The supernatants were used as whole-tissue lysates, and protein concentrations were measured using the BCA protein assay (Bio-Rad, Hercules, CA, USA), according to the instructions provided by the manufacturer. Equal amounts of protein from brain tissue in the middle cerebral artery-supplied region were separated on 10% sodium dodecyl sulfate-polyacrylamide gels for 35 min at 200 V and transferred onto a 0.2 μm PVDF membrane (Thermo Scientific, Hudson, NH, USA) at 70 V for 2 h. The membranes were blocked for 2 h at room temperature with 5% skim milk and washed 3 times with Tris-buffered saline-Tween buffer for 10 min each time. The membranes were then incubated with the primary goat anti-MMP-2 and VEGF (Abcam, Cambridge, MA, USA, 1:1000) overnight at 4 °C. The membranes were washed three times with Tris-buffered saline-Tween buffer, incubated for 2 h with rabbit anti-rat HRP (1:5000; Zhongshanjinqiao, Beijing, China) and washed three times with PBS-Tween. The immunoreactive bands were visualized with the ECL system (Baoxin Biotec, Shenyang, China), and a microplate reader was used to measure the optical density at 540 nm and generate a standard curve. GAPDH was used as a loading control to confirm the equality of total added protein.

### 3.7. CBF Measurements

CBF measurements were performed using the ^14^C-iodoantipyrine technique under etomidate anesthesia (0.03 mg/kg body weight per minute). Rats were infused with ^14^C-iodoantipyrine (125 Ci/kg body weight in 100 μL saline) through the left femoral vein during a 1 min period via a pump at a progressively increasing rate. During the infusion, arterial blood samples, which were freely flowing from the catheterized femoral artery, were collected onto preweighed filter paper disks and subsequently were measured in a liquid scintillation counter. Two minutes later, animals were decapitated, and the brains were removed and snap-frozen in isopentane on dry ice. Sections of 20 mm were prepared, thaw-mounted on glass coverslips, immediately dried on a hot plate and exposed to X-ray films with a set of calibrated ^14^C-polymer standards. In the coronal brain sections corresponding to interaural +5.34 mm regions of interest were selected corresponding to somato-sensory cortex, jaw region. CBF was measured by a computerized image analyzer using an ellipsoid cursor (0.25 ± 2 mm) (MCID Elite, Imaging Research, St. Catharines, ON, Canada). Tissue-blood partition coefficient was defined as 0.7. The software converted the optical density to radioactive content and to CBF using the radioactive standards and the ^14^C-iodoantipyrine blood curve.

### 3.8. Statistical Analysis

SPSS software (Version 20.0) was used for all statistical analyses. The data are presented as mean ± S.E.M. The level of statistical significance was at least *p* < 0.05 throughout this study. Comparisons were made by 1- or 2-way ANOVA or repeated-measures ANOVA, followed by Tukey’s test.

## 4. Conclusions

In conclusion, we demonstrated the effects of running on MMP2 upregulation and VEGF production in rats’ ischemia brain tissue. Our findings suggested a functional interplay between running and these proangiogenic molecules and rCBF. The protective effects of regular exercise on brain function are well recognized. Clearly, physical activity exerts pleiotropic protective effects far beyond purely vascular mechanisms. Both nerve growth factor (NGF) and brain-derived neurotrophic factor (BDNF) have been implicated as downstream mediators. This can, at least in part, be explained by the fact that MMPs facilitates conversion of pro-NGF to NGF and of pro-BDNF to BDNF in the brain, which potentially alter the capacity of neurons and axons to regenerate after nervous system injury [56,57], and MMPs were partially regulated by VEGF, as we have shown.

## Figures and Tables

**Table 1:** Experimental design.

Group	Number	MCAo/R	BEV Treatment	Time for Running (hours post MCAO)	Duration of Running (days)	Timing for Sacrifice after MCAo (days)
1	5	-	-	-	-	0
2	5	+	-	-	-	1
3	5	+	-	48	3	5
4	5	+	-	48	7	9
5	15	+	-	48	14	16
6	5	+	-	-	-	5
7	5	+	-	-	-	9
8	15	+	-	-	-	16
9	10	+	+	48	14	16
10	10	+	+	-	-	16
